# Effect of nanocellulose-based edible coatings enriched with α-pinene and myrtle essential oil on the postharvest quality of strawberry

**DOI:** 10.1186/s12870-025-06757-7

**Published:** 2025-07-02

**Authors:** Somayeh Karimi Dehbakri, Abdollah Ehtesham Nia, Hasan Mumivand, Somayeh Rastegar

**Affiliations:** 1 Department of Horticultural Sciences, Faculty of Agriculture, Lorestan University, Khorramabad, Iran; 2 Department of Horticultural Sciences, Faculty of Agriculture and Natural Resources, University of Hormozgan, Bandar Abbas, Iran

**Keywords:** Strawberry, Coatings, Essential oil, Nanocellulose, Storage, Antioxidant

## Abstract

**Background:**

Despite the desirable qualities of strawberries, their short shelf life and susceptibility to spoilage present significant challenges. This study examined the effect of nanocellulose-based edible coatings enriched with myrtle essential oil (MEO) and α-pinene (AL) at concentrations of 0.3% and 0.6%, on the quality attributes of strawberry fruits during an 18-day storage period at 4 °C. The physico-chemical, nutritional, and sensory attributes of strawberry were evaluated at 0, 6, 12, and 18 days of storage.

**Results:**

Results demonstrated that treated samples displayed lesser weight loss and greater firmness than the control group during storage. Fruits treated with Nano-MEO 0.6% demonstrated minimum weight loss and maximum firmness (1.55 N). The maximum anthocyanin content was observed in the fruits treated with MEO 0.6 %, which was approximately 2.2 times greater than that of the control. Nano-AL 0.6% exhibited the highest ascorbic acid content (85 mg. 100 g^-1^ FW), which was about 4.25 times greater than that of the control (20 mg. 100 g^-1^ FW). On the 18th day of storage, the maximum total phenol content (9.11 mg. g^-1^ FW) was observed in the Nano-MEO 0.6% treatment group, which was significantly different from that in the control (5.64 mg. g^-1^ FW). The maximum flavonoid content was observed in Nano-AL 0.3% (20 mg. g^-1^ FW), while the minimum concentration was found in the control (12.6 mg. g^-1^ FW).The highest catalase (CAT) activity (5.1 U·g⁻^1^ FW) was observed on the 12th day in the Nano-AL 0.6% treatment, whereas the maximum peroxidase (POD) activity (46.7 U·g⁻^1^ FW) was recorded on the same day under the Nano-AL 0.3% treatment. At the end of storage, Nano-MEO (0.6%) exhibited the lowest polyphenol oxidase (PPO) enzyme activity (4.77 U g^-1^ FW), significantly lower than that observed in the control group (13.2 U g^-1^ FW).

**Conclusions:**

In general, compared with the control and other treatments, Nano-MEO (0.6%) demonstrated the greatest overall acceptability. Therefore, the use of this treatment is recommended for maintaining the quality of harvested strawberries.

## Introduction

Strawberry (*Fragaria ×* *Ananassa* Duch.) fruit is widely regarded as an economically significant fruit and is recognized globally for its appealing flavor and taste. They contain a wealth of bioactive components, such as anthocyanin, vitamin E, β-carotene, vitamin Cand which offer various health benefits [1]. Despite their desirable qualities, strawberries are highly perishable fruits with a short storage time. They are susceptible to rapid deterioration due to their high respiration rate, resulting in a shortened shelf life [2]. Additionally, mechanical injuries can lead to the infestation of various mold species, causing notable changes in firmness, color, and overall quality. Factors such as water loss through respiration and transpiration, as well as the development of fungal growth during the ripening process, further contribute to the challenges in improving the freshness and increasing the shelf life of strawberries [3]. Insufficient wax protection on the delicate surface of postharvest strawberries increases their susceptibility to moisture loss, resulting in unfavorable effects such as shriveling, deterioration, and surface color fading. The amount of water lost by fruits is determined by the difference in vapor pressure between the internal tissue of the fruit and the surrounding atmosphere [4]. Therefore, effective postharvest treatments are crucial for maintaining fruit quality during storage. Recently, consumer preferences have shifted towards healthy, more natural and environmentally friendly foods [5].

Recent interest in sustainable methods for extending the shelf life of perishable fruits such as strawberries has led to the development of edible coatings. These coatings slow deterioration by protecting against oxygen, moisture, and microbes. Additionally, incorporating natural additives enhances their effectiveness through antimicrobial and antioxidant properties [6]. Nanocellulose-based edible coatings provide a sustainable and consumer-friendly approach to food preservation. These biodegradable and non-toxic coatings improve shelf life by enhancing barrier properties, managing moisture, and controlling gas permeability. Additionally, nanocellulose can scavenge ethylene, a ripening hormone, thus extending the freshness of produce [7]. It has been reported that guavas coated with sodium alginate-crystalline nanocellulose Pickering emulsions, enriched with thyme and clove essential oils, demonstrated significantly improved retention of ascorbic acid and firmness compared to uncoated controls during a 12-day storage period [8]. In addition, it has been reported that nanocellulose and chitosan-myrtle essential oil extend strawberry fruit storage by 24 days, enhance marketability, and maintain nutritional quality [9]. The incorporation of essential oils into edible coatings could further enhance their protective effects due to their known antimicrobial and antioxidant properties. Myrtle (*Myrtus communis* L.), a member of the Myrtaceae family, is an evergreen shrub characterized by its aromatic white flowers and indigenous to the Mediterranean region. Traditionally employed in folk medicine for its anti-inflammatory and antiseptic effects, recent scientific investigations have focused on its notable antioxidant and antimicrobial properties [10]. Eucalyptol and α-pinene have been recognized as the main compounds existing in the essential oils derived from the leaves of *Myrtus communis* L. [10]. α-Pinene, a naturally occurring monoterpene found in Myrtle and other plants, contributes to its characteristic aroma and possesses potential therapeutic properties such as anti-inflammatory and antimicrobial effects [11, 12]. The chitosan nanoparticles loaded with α-pinene preserved the physicochemical quality of bell pepper [13]. Furthermore, the application of myrtle essential oil was shown to reduce weight loss, decrease the occurrence of decay, and increase the browning index, thereby improving the postharvest storability of fresh loquat fruits [14].

In our previous study, myrtle essential oil was found to be effective at maintaining the quality of strawberry fruits and extending the storage life of strawberries [13]. Therefore, in this study, the effect of nanocellulose edible coatings incorporated with the active component of myrtle essential oil (α-pinene) on strawberries was evaluated. The effects on postharvest quality, including weight loss, firmness, and antioxidant levels, were evaluated over 18 days at 4 °C. The goal is to enhance shelf life and reduce waste, addressing a significant industry challenge.

## Materials and methods

### Sample preparation

Strawberries (*Fragaria × ananassa* cv. ‘Parous’) were harvested at the commercial maturity stage (75% of fruit surface displaying red coloration) from a research greenhouse located in Kordestan, Iran. The harvested fruits were promptly transported to the laboratory for further analysis. Only healthy fruits that were uniformly sized and free from any signs of disease or injury were chosen for further analysis.

### Preparation of essential oil and α-pinene-loaded nanocellulose particles

The nanocellulose gel was procured from Nano Novin Polymer Co., located in Gorgan, Iran. The essential oil and α-pinene were purchased from Khorraman Company (Khorramabad, Iran). First, 1.5 g of Nanocellulose was stirred in 100 mL of distilled water for 40 min on a stirrer. Then, 10 mL of glycerol, essential oil, and α-pinene at concentrations of 0.3% and 0.6% were added. After the solution was sonicated, it was used immediately.

### Fruit treatments

**Fig. 1:**
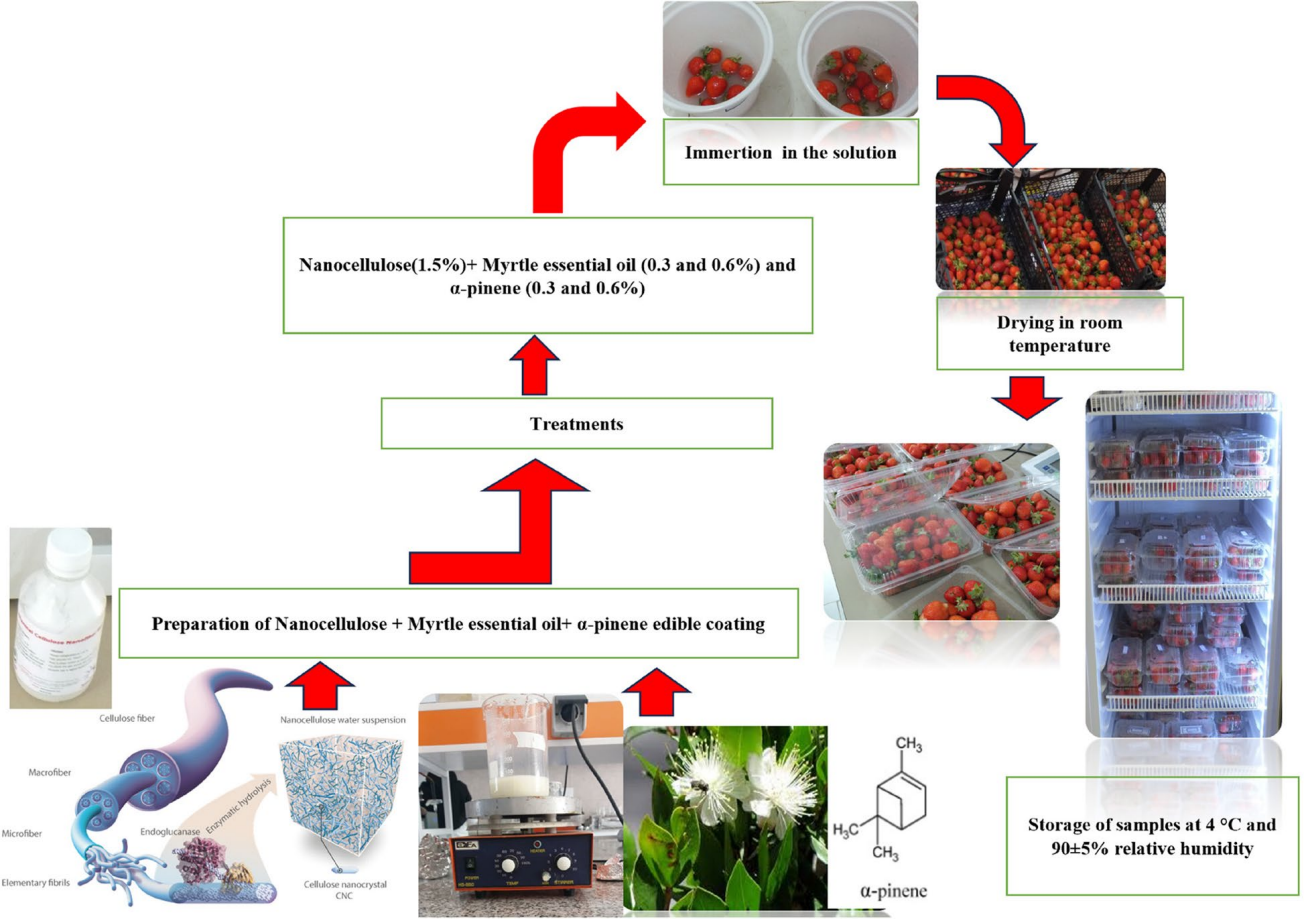
Experimental treatments and their details

Before the experiment, the strawberries were disinfected by soaking them in a 1% (w/v) sodium hypochlorite solution. After that, they were rinsed with purified water and left to air-dry at room temperature for an hour [15]. The experimental fruits were immersed in various treatment solutions for 20 seconds, as detailed in Table 1, and then air-dried at room temperature. The samples were placed in sealed, disposable plastic containers and stored at 4 °C and 90% ± 5% relative humidity for 18 days. Physical and chemical assessments were conducted every 6 days. A summary of the experiment steps shown in Fig. 1.

**Table 1:** Experimental treatments and corresponding abbreviations for edible coatings

Nano	Essential oil	Abbreviation
0	0	Control
	Myrtle essential oil 0.3%	MEO 0.3%
	Myrtle essential oil 0.6%	MEO 0.6%
	α-pinene 0.3%	AL 0.3%
	α-pinene 0.6%	AL 0.6%
1.5	0	Nano 1.5%
	Myrtle essential oil 0.3%	Nano-MEO 0.3%
	Myrtle essential oil 0.6%	Nano-MEO 0.6%
	α-pinene 0.3%	Nano-AL 0.3%
	α-pinene 0.6%	Nano-AL 0.6%

### Weight loss (WL) percentage

The weight loss of the strawberries was determined by calculating the difference between the initial weight (W0) and the weight recorded after each 6-day interval (W1), as described by [15]. The weight loss percentage was then calculated using the following formula:

1 $$\text{WL}=\frac{{w}_{0}-{w}_{1}}{{w}_{0}} \times 100$$

where *w*_0_ represents the initial mass and *w*_1_ represents the final mass.

### Firmness

The firmness of the fruit tissue was measured using a Lutron FG5020 firmness meter. Each fruit was penetrated once with a 3 mm diameter penetrating rod. The firmness of the fruit tissue was determined based on the maximum force required for the rod to penetrate the fruit flesh and is expressed in N [16].

### Measurement of soluble solid content (SSC) and titratable acidity (TA)

The determination of soluble solid content (SSC) was carried out according to the AOAC 932.12 standard using a manual refractometer (ATAGO Company, Fukuoka, Japan) at a temperature of 20 °C. The pH titration method described by Hernandez-Munoz et al. was employed, utilizing a pH meter (HI 9126, Hanna Instruments Inc., Romania) and 0.1 M NaOH until a pH of 8.1 was reached [17].

### Determination of ascorbic acid

A 2,6-dichlorophenolindophenol (DCPIP) titration was employed to assess the ascorbic acid content. A 5 g portion of fruit tissue was homogenized in 50 mL of 0.02 g/ml oxalic acid. After centrifugation, a 10 mL aliquot of the supernatant was titrated with a 0.1% DCPIP solution until a persistent pink coloration was observed. Ascorbic acid concentrations were determined and reported as mg 100 g^−1^ fresh weight [18].

### Determination of total anthocyanin content (TAC)

According the Giusti and Wrolstad [19] method, the fruit was homogenized in a buffer solution containing 0.025 M KCl at pH 1.0. Following a 3-hour heating period, the mixture was centrifuged at 10,000 rpm for 20 min. The resulting supernatant was diluted with a buffer solution comprising 0.025 M KCl at pH 1.0 and 0.4 M sodium acetate (C₂H₃NaO₂) at pH 4.5, according to the required dilution factor. The diluted samples were allowed to equilibrate for 15 min. Absorbance was then measured at 496 nm and 700 nm using a UV1800 spectrophotometer (Mapada Co., Shanghai, China).

### Measurements of total phenol content (TPC)

The total phenol content was determined using the Folin-Ciocâlteu method [18]. For the analysis, 200 µL of the extract was combined with 2 mL of 10% Folin-Ciocâlteu reagent and 4 mL of 1 M sodium carbonate in a test tube. The mixture was then incubated in a dark environment at room temperature for 2 hours. After that, the absorbance of the samples was measured at 765 nm using a spectrophotometer (Mapada Co., Shanghai, China)*.*

### Measurements of total flavonoid content (TFC)

The total flavonoid content (TFC) was measured using a colorimetric method [20]. The TFC was determined using a colorimetric method. Briefly, 1000 µL of strawberry extract was mixed with 500 µL of 5% sodium nitrite solution for 6 min. Subsequently, 500 µL of 10% aluminum nitrate solution was added, followed by 4 mL of a solution containing 4% sodium hydroxide and 70% ethyl alcohol. After standing for 12 min, the absorbance of the mixture was measured at 502 nm***.***

### Radical scavenging activity (DPPH assay)

The total antioxidant capacity was measured using the DPPH (2,2-diphenyl-1-picrylhydrazyl) assay, based on the method described by [19], with some modifications. 2 mL of the methanol extract from the sample was mixed with 2 mL of DPPH solution. DPPH solution was prepared by dissolving 0.25 g of DPPH in 100 mL of 85% methanol (Sigma Aldrich Co., St. Louis, MO, USA). The mixture was vortexed to ensure thorough mixing. The tubes were then left to stand for 30 minutes at room temperature, after which the DPPH radicals were allowed to react with the antioxidants present in the extract. After the incubation period, the absorbance of the reaction mixture was measured against a blank (methanol) at a wavelength of 517 nm using a spectrophotometer (UV1800, Mapada Co., Shanghai, China). The percentage inhibition of free radicals (DPPH) was calculated using the following equation:

2 $$DPPH \left(\%\right)=\frac{{C}_{a}-{S}_{a}}{{C}_{a}}\times 100$$

where C_a_ and S_a_ represent the control absorbance and sample absorbance, respectively.

### Determination of CAT, POD and PPO enzyme activity

To assess the activity of antioxidant enzymes, we extracted and assayed CAT, POD and PPO.

CAT activity was assessed using the method described by Aebi [21]. The reaction blend contained of 400 μL of 25 mM sodium phosphate buffer (pH 7.0), 0.2 mL of extract and 300 μL of 20 mM H₂O₂. The enzymatic activity of CAT on H₂O₂ was measured by the decrease in absorbance at 240 nm using a spectrophotometer. One unit of CAT activity was defined as the change in absorbance with 0.01 at 240 nm per minute [22].

Method Chance and Maehly [23] was used with slight modifications to assess POD activity. Briefly, a 2.0 g fruit was homogenized in a phosphate buffer containing 1 mM polyethylene glycol (PEG) and 4% (w/v) polyvinylpolypyrrolidone (PVPP). Following centrifugation, the supernatant was isolated. The POD activity assay was conducted in a cuvette containing guaiacol, the enzyme solution and hydrogen peroxide. The reaction was monitored spectrophotometrically at 470 nm at 15-second intervals. One unit of POD activity was defined the amount of enzyme capable of causing a 0.01 change per min at 470 nm [23].

To measure PPO activity, a 0.3 g sample was homogenized in 20 mL of phosphate buffer containing 50 mM PEG and 2% w/v PVP while kept in an ice bath. The mixture was then centrifuged at 6000 rpm for 10 min at 4 °C, and the resulting supernatant was collected for the assay. The reaction mixture was prepared by combining 50 μL of 0.02 M pyrogallol and 3000 μL of phosphate buffer (pH 7), incubating it at 35 °C for 5 min. Afterward, 50 μL of the crude extract was added, and the changes in absorbance were recorded at 420 nm. One unit of PPO activity was defined as the amount of enzyme causing an increase in absorbance of 0.001 per minute [24].

#### Overall visual acceptability (OVA)

The fruits’ visual acceptability was subjectively assessed using a five-point scoring system, as outlined in Table 2.

**Table 2:** Visual acceptability scoring criteria

Score	Description
5	Outstanding: Fruits are fresh, firm, and of superior quality with a shiny appearance. No decay, wilting, or discoloration is evident, and the calyx remains green and sturdy.
4	Good: Acceptable and market-ready. Fruits display minimal signs of softness or slight dehydration but retain good overall quality, with the calyx appearing green.
3	Fair: Not suitable for sale but still consumable. Moderate indications of drying, fading color, and shriveling are present, with the fruit nearing over-ripeness. The calyx may look dry or drooping.
2	Poor: Significant deterioration is evident, including extensive shriveling, over-ripeness, and darkened coloration. The fruits are of substandard quality and unfit for eating, with the calyx visibly shriveled.
1	Very poor: Severe decay is present, with fungal growth visible. The calyx appears yellowed, and the fruit is no longer consumable.

The visual acceptability of each fruit was evaluated based on these criteria, and a corresponding score was assigned according to its condition [25].

### Statistical analysis

This study implemented a three-factor factorial experiment, structured as a completely randomized design (CRD), to evaluate the effects of nanocellulose concentration, essential oil type, and storage duration (Table 3). Mean comparisons were conducted using the Least Significant Difference (LSD) test, with statistical significance determined at *P*<0.05, applying by SAS software version 9.4.

**Table 3:** ANOVA results for the effect of storage time, Nanocellulose, and essential oils on various strawberry characteristics

	TA	Firmness	Wight loss	Ascorbic acid	Phenol	Flavonoid	Anthocyanin	DPPH	CAT	POD	PPO
T	**	**	**	**	**	**	**	**	**	**	**
A	**	*	**	**	ns	ns	**	ns	**	**	**
B	**	ns	**	**	**	**	**	**	*	**	ns
T × A	**	*	**	**	**	**	**	ns	**	**	ns
T × B	**	**	**	**	**	**	**	**	**	**	**
A × B	**	*	**	**	**	**	**	**	**	**	**
T × A × B	**	ns	**	**	**	**	**	**	**	**	**

T= Storage time, A= Nanocellulose, B =Essential oils

NS not significant, *significant at *P*<0.05, **significant at *P*<0.01

## Results and discussion

### Weight loss

**Fig. 2:**
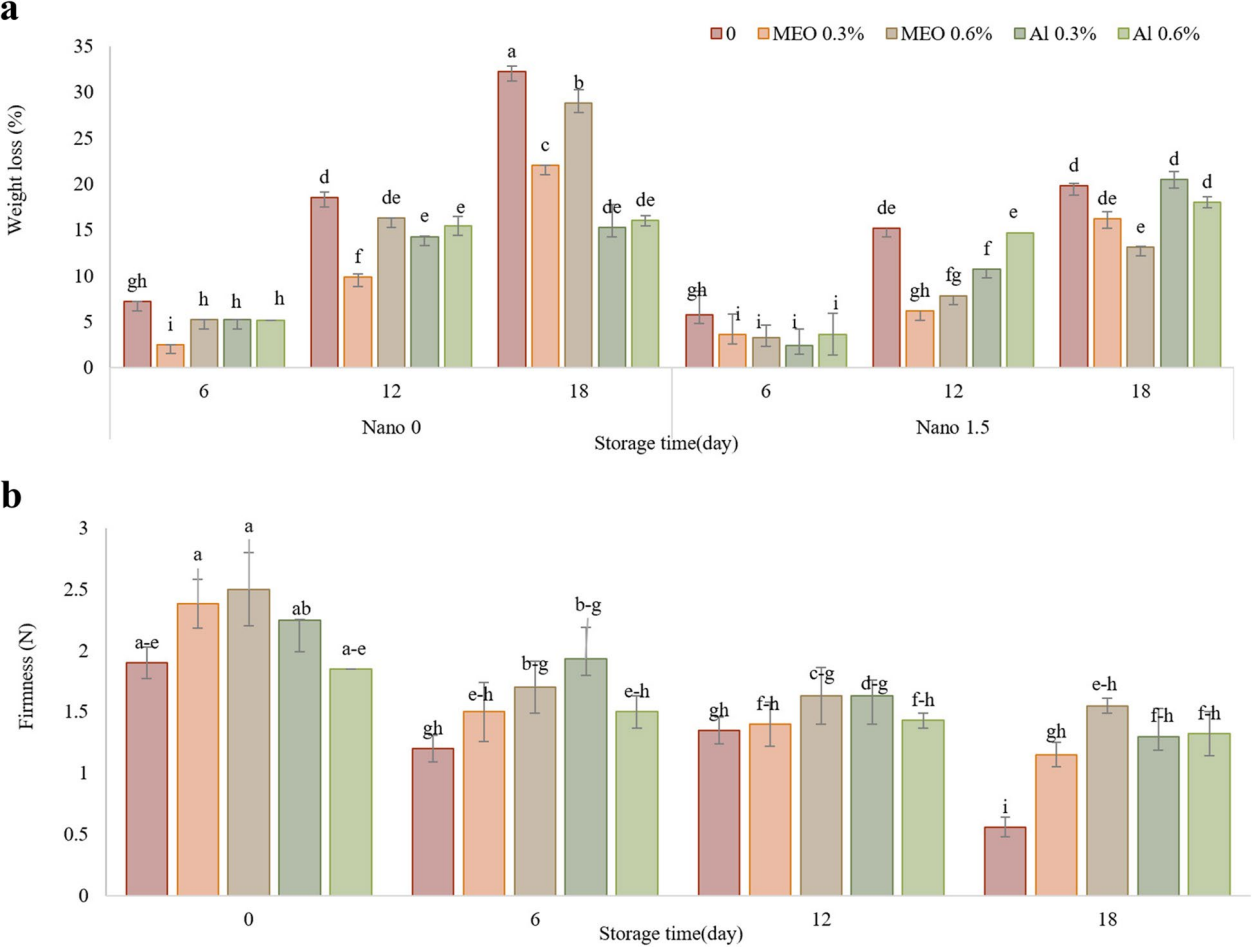
The impact of treatments (Control, MEO 0.3%, MEO 0.6%, AL 0.3%, AL 0.6%, Nano 1.5%, Nano-MEO 0.3%, Nano-MEO 0.6%, Nano-AL 0.3%, Nano-AL 0.6%) on the weight loss (**a**) and firmness (**b**) of strawberry fruit stored for 18 days at 4 °C. The data represent the mean values of *n* = 3, and the error bars indicate standard errors (SE) of the means. Statistical analysis was performed using the LSD test at the *P* ≤ 0.05 level

As depicted in Fig. 2A, there was a gradual increase in weight loss during storage. Nevertheless, the utilization of essential oil either alone or in conjunction with Nanocellulose led to reduced levels of weight loss in comparison to the control group. Specifically, on the 12 th and 18 th days of storage, the control group displayed the greatest weight loss, at 18.5% and 32.2%, respectively. Conversely, the sample treated with Nano-MEO 0.3% (6.17) in the 12^th^ cycle and Nano-MEO 0.6% (13.15%) exhibited the lowest weight loss, highlighting the substantial influence of the combined Nanocellulose and essential oil treatment. Weight loss is a vital factor in evaluating the effectiveness of fruit conservation. As fruits ripen and undergo transpiration, there is a gradual decrease in water content, leading to desiccation and a decrease in mass. Strawberries, characterized by their fragile skin structure, are particularly susceptible to water loss via evaporation and respiration [26]. In the present study, minimal weight loss was observed in the Nano-MEO 0.6% treatment group. Utilizing polysaccharide coatings with the addition of essential oils offers a way to preserve the moisture and desired sensory characteristics of fresh produce [27]. Previous research has indicated that the encapsulation of α-pinene within chitosan Nanoparticles has the potential to decrease the weight loss of bell pepper during a 21-day storage period at 12 ± 2 °C [13]. Similarly, in a study by Lee et al., strawberries treated with a sodium alginate, bagasse cellulose nanocrystal, chitosan nanofiber and oregano essential oil formulation exhibited a weight loss of only 10.8 % after nine days of storage, which was lower than that of uncoated strawberries, whose weight loss exceeded 37.0 % [28].

### Firmness

The interaction between essential oils, Nanoparticles, and storage time did not significantly affect fruit firmness (Table 3). Therefore, the individual effect of time on fruit firmness was assayed. According to Fig. 2B, fruit firmness gradually decreased over time. In the control group, the firmness decreased from an initial measurement of 1.6 N to 0.56 N. Conversely, the firmness of the treated samples diminished less markedly. Consequently, on day 18 of storage, the firmness of the treated samples was greater than that of the control group. Moisture loss and high respiration rates during storage contribute to a decrease in strawberry fruit firmness, which is a crucial factor for its marketability [29]. During the ripening process, the firmness of fruits decreases because of enzymatic activity, specifically the conversion of insoluble pectic fractions into soluble forms by enzymes such as protopectinase and pectin methyl esterase. Polygalacturonase, another enzyme, plays a crucial role in transforming pectic polysaccharides into water-soluble forms [30]. In addition to enzymatic changes, softening in fruits can also occur due to water loss, resulting in a loss of turgor and crispness [31]. Recently, a study demonstrated the effectiveness of volatile essential oils derived from myrtle leaves in preventing changes in the firmness of loquat fruits [14]. Research has indicated that the application of multi-polysaccharide coating suspensions results in the formation of a thin layer on the surface of strawberries. This layer serves as a barrier, restricting transpiration and gas exchange through the lenticels on the fruit. Consequently, it slows down respiration and delays fruit softening [28]. According to Lee et al. [28], the coated strawberries demonstrated superior firmness retention during storage, retaining more than 40 % of their initial firmness after nine days. In contrast, the uncoated strawberries experienced a significant loss of firmness, with a nearly 90 % reduction during the same storage period. The application of encapsulated oregano essential oil at a concentration of 2 % resulted in significantly greater firmness in pear fruit compared to both the control group and the treatments using pure oregano essential oil [32]. Cellulose Nanofibers exhibit favorable wettability due to the presence of numerous hydroxyl (–OH) groups on their surface. These hydroxyl groups can form stable hydrogen bonds with water (H_2_O), effectively “locking” it onto the surface of food [33].

### TSS and TA

According to Table 4, fruits treated with essential oil displayed greater acidity than fruits subjected to the other treatments. Specifically, fruits treated solely with essential oil, except for those treated with 0.6% AL, exhibited higher acidity levels than those in the control group. During the storage period, the total soluble solids (TSS) content of the fruits gradually increased. This increase was more pronounced in the control fruits, indicating a greater degree of ripening. In contrast, compared with those in the control group, the TSS content in the treated fruits decreased.

**Table 4:** The impact of treatments (Control, MEO 0.3%, MEO 0.6%, AL 0.3%, AL 0.6%, Nano 1.5%, Nano-MEO 0.3%, Nano-MEO 0.6%, Nano-AL 0.3%, Nano-AL 0.6%) on the TA (%) and TSS of strawberry fruit stored for 18 days at 4 °C. The data represent the mean values of *n* = 3, and the error bars indicate standard errors (SE) of the means. Statistical analysis was performed using the LSD test at the *P* ≤ 0.05 level

Parameters	Storage time(day)	Control	EO 0.3%	EO 0.6%	Al 0.3%	Al 0.6%	Nano-1.5%	Nano-EO 0.3%	Nano-EO 0.6%	Nano-Al 0.3%	Nano-Al 0.6%
TA (%)		0.9^a^	0.9^a^	0.9^a^	0.9^a^	0.9^a^	0.9^a^	0.9^a^	0.9^a^	0.9^a^	0.9^a^
	0										
		0.51^jklmn^	0.65^efghi^	0.75^bdce^	0.72^bcdef^	0.76^bdce^	0.51^jklmn^	0.83^ab^	0.69^defgh^	0.81^abc^	0.78^bdc^
	6										
		0.7^cdefg^	0.8^abcd^	0.75^bdce^	0.72^bcdef^	0.76^bdce^	0.70^cdefg^	0.75^bcde^	0.8^abcd^	0.76^bdce^	0.8^abcd^
	12										
		0.45^mn^	0.61^fghij^	0.61^fghij^	0.61^fghij^	0.3^o^	0.60^jhij^	0.45^mn^	0.44^n^	0.46^mn^	0.48^klmn^
	18										
TSS (Brix)		9.4^cd^	9.33^cd^	9.50 ^cd^	9.50 ^cd^	9.50 ^de^	9.5 ^cd^	8.9 ^e^	9.5 ^bcd^	9^e^	9^e^
	0										
		10^c^	10^c^	9^e^	10^c^	9^e^	9.5^cd^	10^c^	9^e^	8.50^ef^	10^c^
	6										
		10.3^bc^	9.4^cd^	10^c^	10^c^	10^cde^	10^cde^	9.8^cd^	10^c^	11^a^	9.6^cde^
	12										
		11^a^	10.2^bc^	10^c^	10.5^b^	10^c^	10^c^	10.6^b^	10^c^	10.5^b^	10.7^ab^
	18										

Intermediate metabolites such as organic acids play a crucial role in various physiological reactions within the cell, and they are utilized or consumed as part of these processes. The acidity and TSS content are crucial factors contributing to the overall flavor and quality of fruits [34]. During the storage of strawberries, the TSS content can increase due to several factors. These include the conversion of starch into sugars, breakdown of polysaccharides in the cell wall through hydrolysis, coupled with an increase in sugar concentrations due to moisture loss [35]. The findings from the treatment showed that the fruit acidity reduced as the storage time progressed. This result demonstrated that the TA of all strawberries diminished after a period of storage, which aligns with the findings of previous studies [36]. It has been shown that the coating of blackberry fruits with Nanocellulose and essential oil Nanoparticles can have an impact on the soluble solids content, with higher concentrations of Nanocellulose potentially leading to a reduction in soluble solids during storage [37]. Throughout the storage period, the respiration rate of the fruits consistently increased, leading to the decomposition of a substantial amount of organic matter into sugars, acids, and minerals. As a result, the concentration of soluble solids also increased. In general, the application of a multilayer coating effectively restrained the increase in soluble solids during storage. Despite better preservation potential, the effect of essential oil-based edible coatings on the sensory properties of fruits is one of the major aspects that affects consumer decisions during purchase [38].

### Ascorbic acid and total anthocyanin content (TAC)

**Fig. 3:**
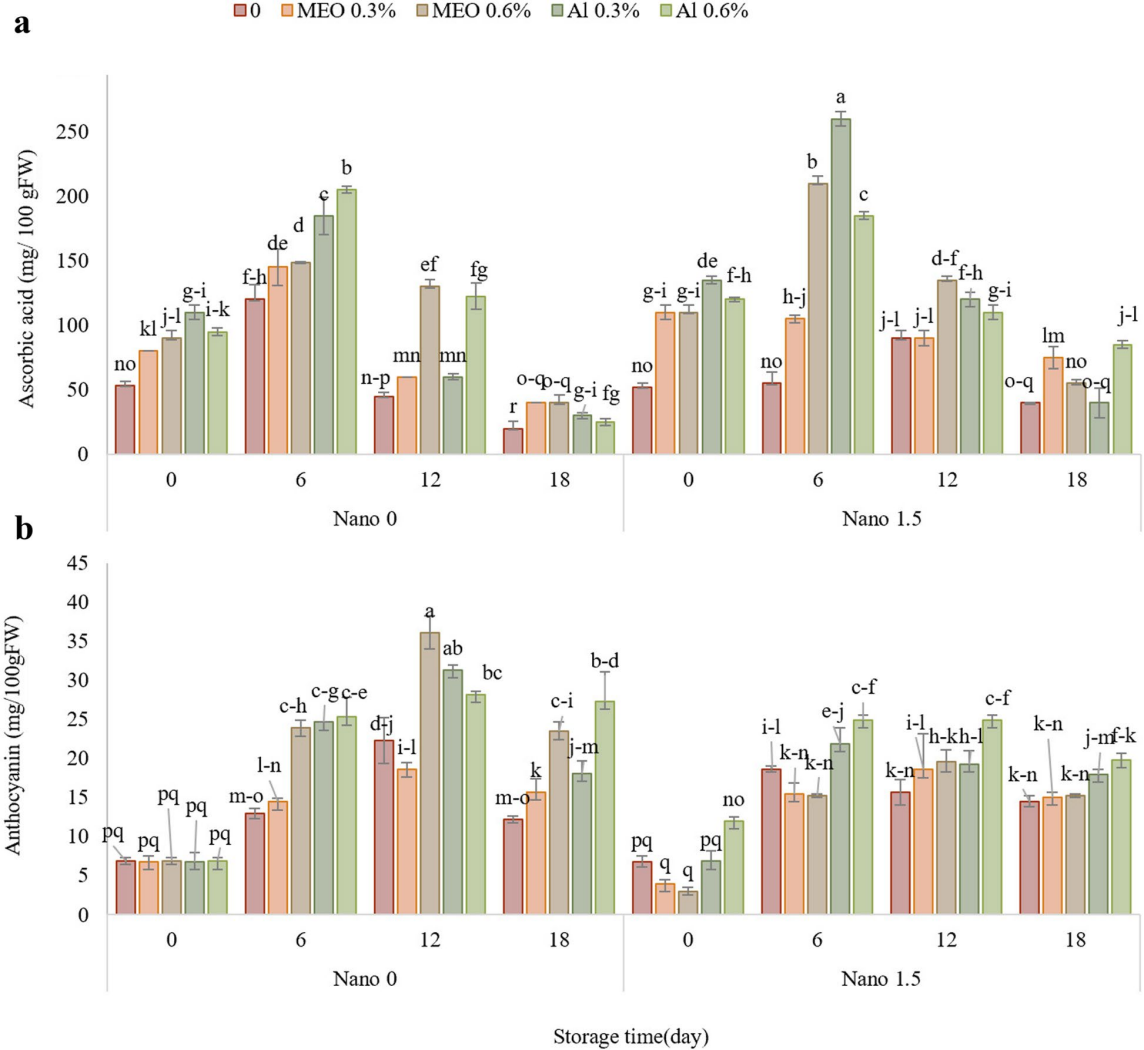
The impact of treatments (Control, MEO 0.3%, MEO 0.6%, AL 0.3%, AL 0.6%, Nano 1.5%, Nano-MEO 0.3%, Nano-MEO 0.6%, Nano-AL 0.3%, Nano-AL 0.6%) on the ascorbic acid (**a**) and anthocyanin (**b**) of strawberry fruit stored for 18 days at 4 °C. The data represent the mean values of *n* = 3, and the error bars indicate standard errors (SE) of the means. Statistical analysis was performed using the LSD test at the *P* ≤ 0.05 level

During the storage period, the ascorbic acid content initially increased on day 6 and then gradually decreased (Fig. 3A). On day 18 of storage, the control group exhibited the lowest ascorbic acid content, at 20.2 mg. 100 g^−1^, which was significantly different from that of the other treatment groups. The composite samples containing Nanocellulose subjected to various treatments displayed higher levels of ascorbic acid, indicating the significant effect of Nanocellulose in enhancing the effectiveness of MEO and AL.

The anthocyanin concentration gradually increased until day 12 of storage and then decreased by day 18. At the end of the experiment, the samples treated with AL 0.6% (27.28 mg. 100 g^−1^ FW) exhibited the highest anthocyanin content, while the control group displayed the lowest anthocyanin content (Fig. 3B).

During the experiment, strawberries treated with Nano-AL exhibited a significantly greater antioxidant content than both the control group and the other treatment groups. Edible coatings help maintain the ascorbic acid content in fruits by creating a barrier that reduces oxygen and moisture transfer, which are factors that contribute to the degradation of ascorbic acid during storage [6]. The chitosan/cinnamaldehyde Nanocomposite film showed potential for preserving the ascorbic acid content of mangoes during storage [39]. Coating materials such as edible films can provide a barrier that prevents exposure to oxygen, light, and moisture, which are factors that can degrade ascorbic acid [40].

Edible coatings can also influence the stability and retention of anthocyanins in food products. Anthocyanins are sensitive to factors such as light, oxygen, and pH, which can lead to their degradation [41]. In the present study, fruits treated with a high concentration of essential oil and α-pinene, as well as those treated with Nano-MEO and Nano-AL, exhibited the highest anthocyanin content. Coating can protect anthocyanins from unfavorable conditions, such as pH, temperature, light, oxygen, and enzymes, which can affect their stability [42]. These coatings can also provide a physical barrier that prevents the migration of anthocyanins from the food matrix to the surrounding environment. By reducing the exposure of anthocyanins to external factors, edible coatings can help to preserve their color and antioxidant properties. Additionally, edible coatings can enhance the bioavailability of anthocyanins by improving their solubility and absorption in the gastrointestinal tract [43]. In line with our findings, strawberries that were treated with a carboxymethylcellulose-based edible coating enriched with essential oil demonstrated elevated levels of anthocyanin content compared to untreated samples throughout the storage period. The application of the coating likely protected the fruit by inhibiting the enzymatic oxidation of compounds, thus potentially influencing the anthocyanin levels in the strawberries [44]. In addition, strawberry samples that were coated with an antioxidant extract and bergamot essential oil exhibited reduced decay rates and superior preservation of anthocyanin content following a 14-day storage period. The coatings created favorable conditions that prolonged the storage time compared to that of the control sample, thereby delaying the onset of ripening and senescence. As a result, antioxidant parameters, such as the anthocyanin content, were effectively preserved [36].

### Total phenol and flavonoid contents and antioxidant capacity

**Fig. 4:**
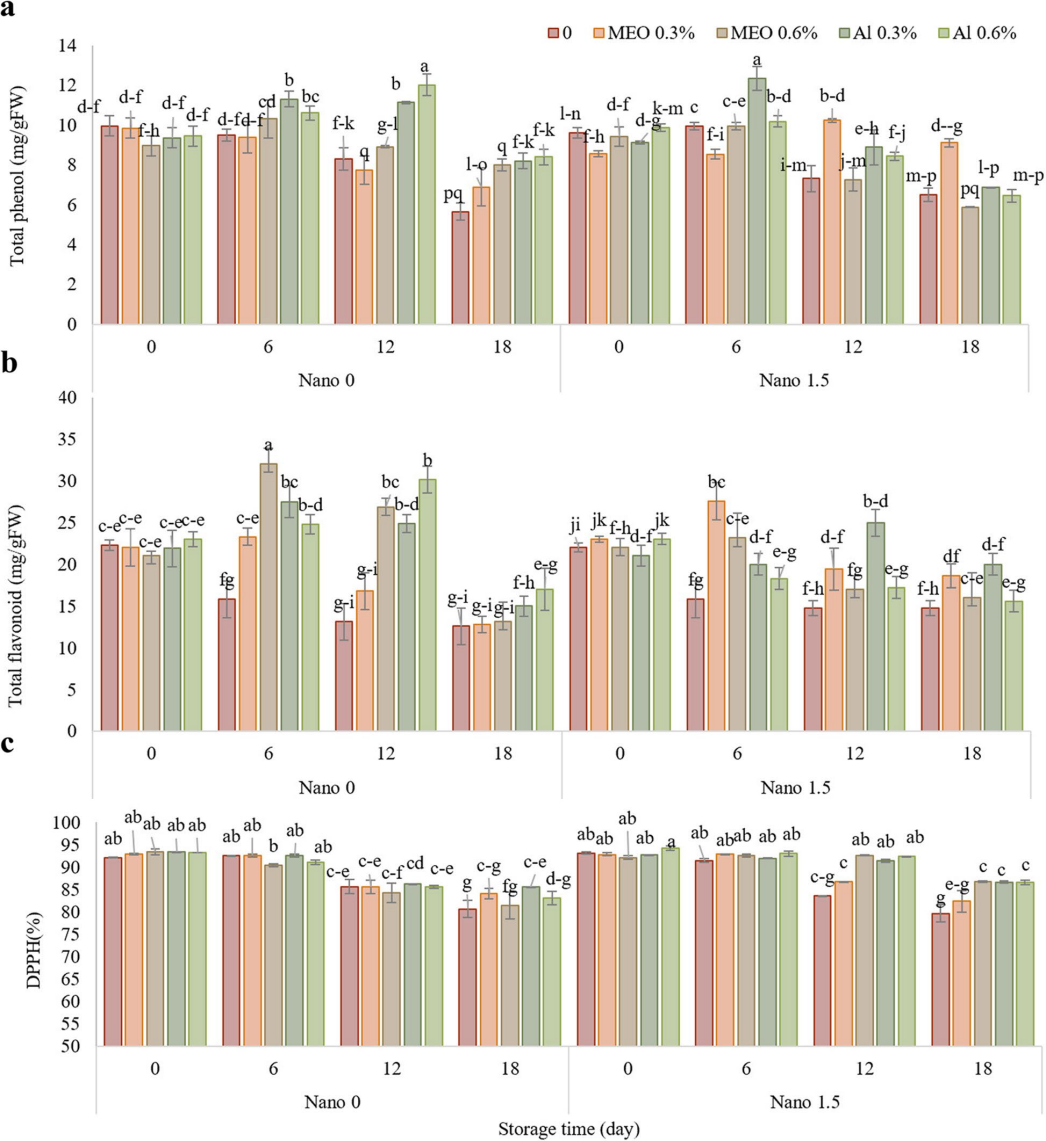
The impact of treatments (Control, MEO 0.3%, MEO 0.6%, AL 0.3%, AL 0.6%, Nano 1.5%, Nano-MEO 0.3%, Nano-MEO 0.6%, Nano-AL 0.3%, Nano-AL 0.6%) on the total phenol (**a**) total flavonoid (**b**) and antioxidant capacity (**c**) of strawberry fruit stored for 18 days at 4 °C. The data represent the mean values of *n* = 3, and the error bars indicate standard errors (SE) of the means. Statistical analysis was performed using the LSD test at the *P* ≤ 0.05 level

As illustrated in Fig. 4 A, the phenol content in the control samples gradually decreased and reached its lowest point on the 18 th day. Compared with the control, the treated samples had a greater phenol content. Consequently, on the 18 th day of storage, the samples treated with Nano-MEO 0.3% exhibited the highest phenol content (9.11 mg. g^−1^ FW).

The flavonoid content of the control also decreased significantly during storage, reaching its minimum on the 18 th day. In certain treated samples, the flavonoid content initially increased and then decreased. Ultimately, at the end of the experiment, the Nano-AL 0.3% The treatment had the greatest flavonoid content (19.99 mg. g^−1^ FW), followed by the Nanocellulose-essential oil treatment.

Regardless of the treatment employed, the antioxidants in the samples displayed minimal changes at the onset of storage but exhibited a substantial decrease by the end of storage. The samples treated with MEO 0.6%, Nano-AL 0.3% and Nano-AL 0.6% showed the highest (86.6%) antioxidant levels. Conversely, the control treatment exhibited the lowest antioxidant capacity (79.66%).

Phenolic compounds play a crucial role in providing both sensory and nutritional properties to various fruits. However, during the storage period, the oxidation of phenolic components occurs due to the action of polyphenol oxidase, resulting in the formation of dark-colored pigments. This oxidation process leads to a reduction in the overall antioxidant activity of the fruits [45]. Furthermore, the degradation and decrease in phenolic content are closely linked to the breakdown of cell structure and the ongoing senescence of fruits. However, in the current study, the application of Nanocellulose edible coatings containing essential oils has the potential to mitigate this decline. Edible coatings have been shown to preserve important compounds such as flavonoids, phenolics, carotenoids, lycopene, and glucosinolates, thereby reducing the deterioration of fruits [46]. Specifically, the phenolic compounds in fruits are susceptible to oxidation, which can lead to a loss of color, flavor, and nutritional value. Essential oils can help to prevent this oxidation by scavenging free radicals and inhibiting enzymes that promote oxidation [47]. The study showed that the controlled delivery of *Citrus sinensis* essential oil through a Nanoemulsion system preserved the antioxidant activity of avocado fruits by efficiently and effectively delivering the essential oil to the fruit surface [48]. In a study by Wang et al. [33], treating apple slices with cellulose Nanofibers increased the levels of total phenols and total flavonoids during storage. The treatment activated the antioxidant defense mechanisms, enhanced the capacity to eliminate ROS, and mitigated membrane lipid peroxidation in the apple slices, thereby demonstrating a protective role in preserving their phenolic compounds***.***

### Enzyme activities

**Fig. 5:**
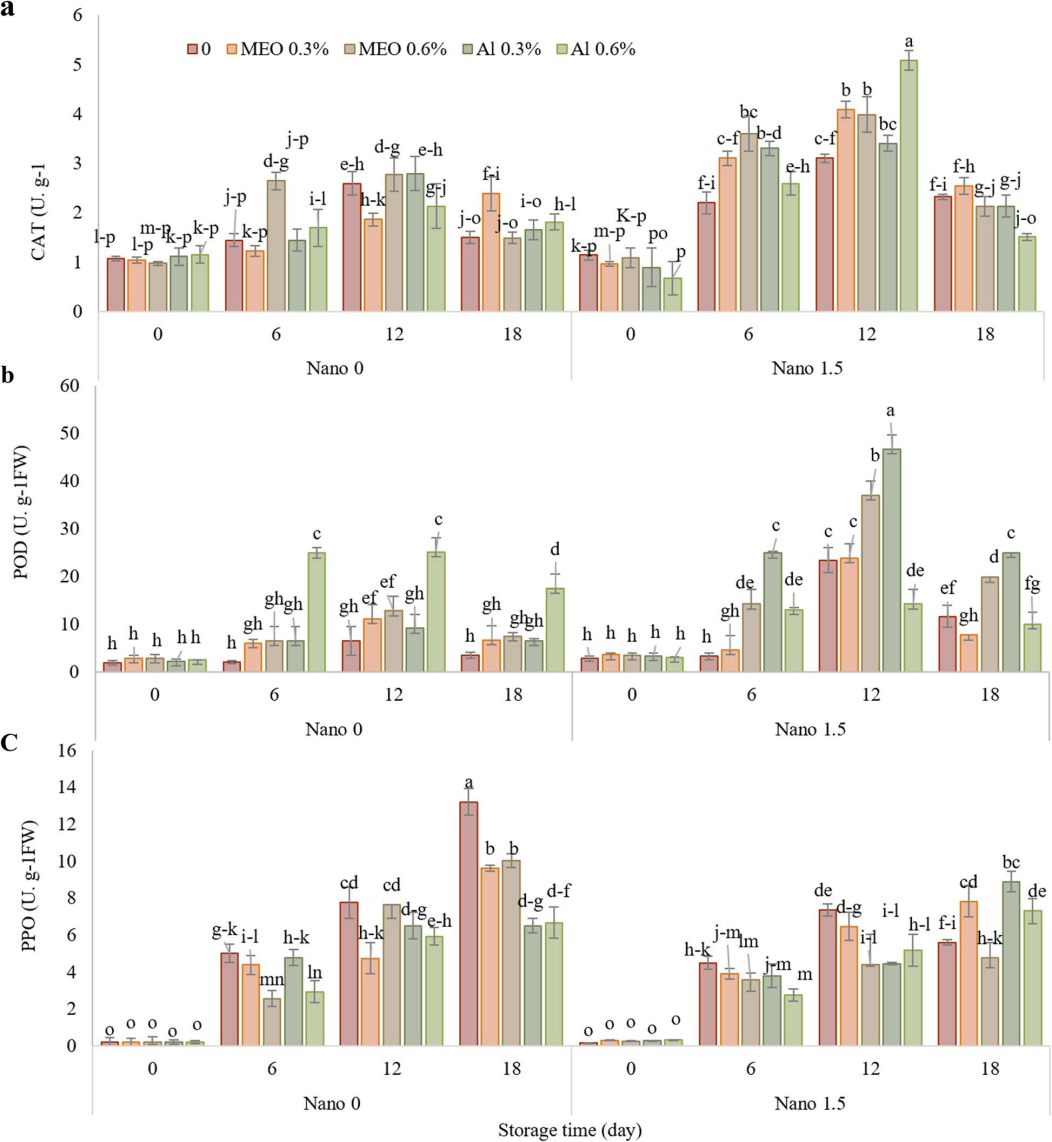
The impact of treatments (Control, MEO 0.3%, MEO 0.6%, AL 0.3%, AL 0.6%, Nano 1.5%, Nano-MEO 0.3%, Nano-MEO 0.6%, Nano-AL 0.3%, Nano-AL 0.6%) on the CAT (**a**), POD (**b**) and PPO activities (**c**) of strawberry fruit stored for 18 days at 4 °C. The data represent the mean values of *n* = 3, and the error bars indicate standard errors (SE) of the means. Statistical analysis was performed using the LSD test at the *P* ≤ 0.05 level

As illustrated in Fig. 5A, the activity of the CAT enzyme gradually increased until day 12 of preservation. However, generally, it decreased on day 18 of preservation. Notably, the fruits treated with 1.5% Nano in combination with essential oil and α-pinene displayed the highest enzyme activity, indicating a significant difference compared to that of the control group. Specifically, the fruits treated with Nano-AL 0.6% exhibited the highest enzyme activity on day 12.

Figure 5B illustrates that POD activity exhibited an initial increase followed by a slight decline during storage. The highest enzyme activity was observed in samples treated with nano in combination with Nano-AL 0.3% and Nano-MEO 0.6%. Among the non-nano treatments, the samples treated with AL 0.6% showed the significantly higher activity during storage.

The activity of PPO also gradually increased during the storage period (Fig. 5C). However, the rate of increase in the treated samples was minor than that in the control group. On the 18 th day of storage, the control group exhibited the highest enzyme activity, while the samples treated with Nano-MEO 0.6% (4.77 U/g FW) exhibited the lowest enzyme activity. Although MEO 0.3% and MEO 0.6% alone displayed lower enzyme activity than the control, they significantly controlled the enzyme activity when combined with Nanocellulose.

During postharvest storage, the accumulation of toxic free radical molecules can lead to a significant loss of nutritional quality in fruits. However antioxidant defense enzymes play crucial roles in scavenging ROS and maintaining the freshness and antioxidant capacity of fruits [49]. CAT and POD are vital elements of the antioxidant defense mechanism in fruits during postharvest storage, primarily responsible for mitigating oxidative stress by eliminating accumulated ROS. CAT’s specific function involves decomposing hydrogen peroxide (H₂O₂) into water and oxygen, thus protecting fruit tissue cells from H₂O₂ toxicity and prolonging their shelf life [50]. POD complements CAT by also catalyzing the conversion of H₂O₂ to water and oxygen, while additionally oxidizing phenolic substrates, thereby removing both H₂O₂ and phenolic toxicity from plant tissues [51]. Beyond its antioxidant role, POD is crucial for maintaining the structural integrity of cell walls and lignin formation, which contributes to fruit firmness during storage [52]. Fruits with higher levels of these enzymes are more effective in reducing ROS accumulation, thereby minimizing oxidative damage, delaying senescence, and extending storage life [49]. These enzymes act as both indicators and protectors of fruit quality during storage. Numerous studies have demonstrated a correlation between higher CAT and POD activities and enhanced fruit quality parameters. Therefore, maintaining high levels of CAT and POD enzyme activities is essential for managing fruit senescence during storage, as these enzymes can scavenge excess ROS, delay the loss of membrane function, and alleviate oxidative stress in postharvest fruits [53]. The current study revealed that samples treated with Nano-MEO and Nano-AL exhibited increased activity of the CAT enzyme. Wang et al. [33] suggested that the rapid increase in ROS levels during storage might be associated with a decline in the activity of antioxidant enzymes and the content of nonenzymatic antioxidants. In a study conducted by Yang et al. [54], it was observed that a Nanoemulsion-based coating containing carvacrol, eugenol, and cinnamaldehyde applied to citrus fruits had a positive impact on the activity of antioxidant defense enzymes. After a storage period of 40 days, the coating led to an increase of 22.29% in CAT activity, 51.49% in POD activity, and 18.12% in SOD activity. These findings highlight the effectiveness of such Nanoemulsion coatings in enhancing the antioxidant defense system of fruits during storage [54].

PPO is an enzyme that significantly contributes to the browning of fruits and vegetables when they sustain damage. Its primary function is to catalyze the oxidation of o-catechol into o-quinone [55]. In the present study, the application of Nano-MEO effectively regulated the activity of the PPO enzyme. Similar to our results, Wang et al. showed changes in PPO enzyme activity during storage, while cellulose Nanofiber-treated apple wedges maintained low PPO activity [33].

### Overall acceptability

**Fig. 6:**
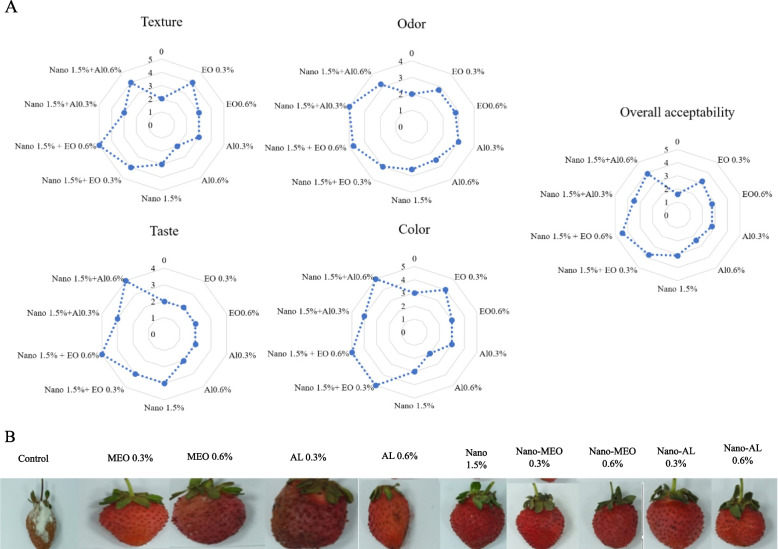
The impact of treatments (Control, MEO 0.3%, MEO 0.6%, AL 0.3%, AL 0.6%, Nano 1.5%, Nano-MEO 0.3%, Nano-MEO 0.6%, Nano-AL 0.3%, Nano-AL 0.6%) on the visual quality and overall acceptability of strawberry fruit stored after 18 days at 4 °C (**A** and **B**)

Based on the observations presented in Fig. 6a, it is apparent that the Nano-MEO 0.6% treatment outperforms both the other treatments and the control group in terms of various textural attributes, odors, and taste properties. The control group displayed noticeable inferiority in terms of fruit quality. Importantly, treatments combining Nanocellulose with essential oil demonstrate superior quality compared to treatments involving essential oil alone (Fig. 6b).

In the present study, the fruits treated with Nano-MEO 0.6% or Nano-AL 0.6% exhibited better overall visual acceptability than the control and other treatments. In addition to the rich presence of hydroxyl groups, the gas barrier property of Nanocellulose was identified as one of the main contributing factors to the favorable outcome in terms of visual retention [56]. The positive effects of adding essential oils to edible coatings have been mentioned in other studies. According to a study conducted by [47], fruits treated with essential oils exhibited greater acceptance and sensory qualities than untreated fruits after storage. Research has demonstrated that the application of citrus essential oils can enhance the freshness and aroma of strawberries, leading to improved acceptance and sensory attributes. Coating suspensions form a thin protective layer on the surface of the fruit, effectively reducing moisture loss, preventing microbial contamination, and minimizing minor mechanical damage [4]. Compared to traditional single-polysaccharide coatings, coating suspensions incorporating sustainable nanomaterials combined with multiple polysaccharides can create darker networks on fruit surfaces, offering superior barrier properties to inhibit the passage of various pathogenic molecules [57]. The cellulose nanocrystal-gelatin coating effectively reduced fruit weight loss and deterioration while preserving the ascorbic acid content of the strawberries [58].

## Conclusion

This study investigated the effects of treatment with myrtle essential oil (MEO), α-pinene (AL), and Nanocellulose (Nano) on the quality of strawberries during storage. The quality of the treated samples improved compared to that of the control group. Fruits treated with Nano-MEO 0.6% exhibited significantly reduced weight loss and enhanced firmness, correlating with the lowest PPO activity. Conversely, treatments with Nano-AL 0.6% and Nano-AL 0.3% displayed the highest CAT and POD activities, respectively. Furthermore, the Nano-AL 0.3% treatment resulted in elevated ascorbic acid and anthocyanin levels at the end of the storage period. Overall, the Nano-MEO 0.6% treatment had the highest overall acceptability. These findings have implications for extending the shelf life of strawberries and reducing their perishability, which can benefit both producers and consumers in the fruit industry.

## Data Availability

The data that support the findings of this study are available from the corresponding author upon reasonable request.
